# Correction: Zhu et al. Gait Analysis with Wearables Is a Potential Progression Marker in Parkinson’s Disease. *Brain Sci.* 2022, *12*, 1213

**DOI:** 10.3390/brainsci13101429

**Published:** 2023-10-08

**Authors:** Sha Zhu, Zhuang Wu, Yaxi Wang, Yinyin Jiang, Ruxin Gu, Min Zhong, Xu Jiang, Bo Shen, Jun Zhu, Jun Yan, Yang Pan, Li Zhang

**Affiliations:** 1 Department of Geriatric Neurology, The Affiliated Brain Hospital of Nanjing Medical University, Nanjing 210029, China; neuro_zhusha@stu.njmu.edu.cn (S.Z.); wangyaxi1102@stu.njmu.edu.cn (Y.W.); neuo_jiangyinyin@stu.njmu.edu.cn (Y.J.); grx@njmu.edu.cn (R.G.); neuro_zhongmin@163.com (M.Z.); jx13773958367@sina.com (X.J.); 15951758927@163.com (B.S.); njmuzhangli@sina.com (J.Z.); kdzb666@163.com (J.Y.); neuro_panyang@163.com (Y.P.); 2 Neurotoxin Research Center of Key Laboratory of Spine and Spinal Cord Injury Repair and Regeneration of Ministry of Education, Neurological Department of Tongji Hospital, School of Medicine, Tongji University, Shanghai 200092, China; 2111723@tongji.edu.cn

## Error in Table

In the original publication [1], there was a mistake in Table 3. After the authors calculated the data for rows 11–16 in column 6 of the table, they read the wrong column when entering, and an error occurred. The corrected Table 3 appears below. The authors state that the scientific conclusions are unaffected. This correction was approved by the Academic Editor. The original publication has also been updated.

Line 11: change −0.431 (<0.001) to −0.441 (<0.001)

Line 12: change −0.382 (<0.001) to −0.375 (<0.001)

Line 13: change −0.429 (<0.001) to −0.434 (<0.001)

Line 14: change 0.517 (<0.001) to −0.332 (<0.001)

Line 15: change −0.454 (<0.001) to −0.246 (0.004)

Line 16: change −0.171 (<0.001) to −0.067 (0.434)

## Figures and Tables

**Table 3:** Correlation analysis between gait parameters and H–Y stage, UPDRS scores and PDQ39.

	H–Y Stage	UPDRS Ⅰ	UPDRS Ⅱ	UPDRS Ⅲ	PDQ39
SL (m)	**−0.566 (<0.001)**	−0.155 (0.070)	**−0.453 (<0.001)**	**−0.454 (<0.001)**	**−0.433 (<0.001)**
GV (m/s)	**−0.458 (<0.001)**	−0.040 (0.644)	**−0.334 (<0.001)**	**−0.376 (<0.001)**	**−0.269 (0.001)**
CA (steps/min)	−0.052 (0.545)	**0.212 (0.012)**	0.001 (0.987)	−0.069 (0.421)	0.110 (0.201)
ST (s)	0.055 (0.518)	**−0.210 (0.013)**	0.003 (0.970)	0.075 (0.383)	−0.109 (0.204)
StPT (%)	**0.247 (0.003)**	−0.073 (0.396)	**0.199 (0.019)**	**0.229 (0.007)**	0.092 (0.284)
SwPT (%)	**−0.268 (0.001)**	0.057 (0.504)	**−0.216 (0.011)**	**−0.253 (0.003)**	−0.062 (0.469)
CV–SL	**0.552 (<0.001)**	**0.219 (0.010)**	**0.500 (<0.001)**	**0.463 (<0.001)**	**0.358 (<0.001)**
CV–ST	**0.401 (<0.001)**	**0.194 (0.023)**	**0.369 (<0.001)**	**0.371 (<0.001)**	**0.224 (0.008)**
CV–StPT	**0.380 (<0.001)**	0.153 (0.074)	**0.315 (<0.001)**	**0.311 (<0.001)**	**0.229 (0.007)**
CV–SwPT	**0.455 (<0.001)**	0.107 (0.211)	**0.373 (<0.001)**	**0.346 (<0.001)**	**0.252 (0.003)**
walk radio	**−0.431 (<0.001)**	**−0.263 (0.002)**	**−0.380 (<0.001)**	**−0.343 (<0.001)**	**−0.441 (<0.001)**
ROM–AJ (°)	**−0.382 (<0.001)**	**−0.250 (0.003)**	**−0.287 (0.001)**	**−0.281 (0.001)**	**−0.375 (<0.001)**
ROM–KJ (°)	**−0.429 (<0.001)**	−0.107 (0.210)	**−0.327 (<0.001)**	**−0.340 (<0.001)**	**−0.434 (<0.001)**
ROM–HJ (°)	**−0.517 (<0.001)**	−0.118 (0.168)	**−0.408 (<0.001)**	**−0.422 (<0.001)**	**−0.332 (<0.001)**
HS (°)	**−0.454 (<0.001)**	−0.133(0.121)	**−0.398 (<0.001)**	**−0.379 (<0.001)**	**−0.246 (0.004)**
TO (°)	**−0.171 (0.044)**	−0.089 (0.302)	−0.105 (0.220)	**−0.144 (<0.001)**	−0.067 (0.434)
